# Stable isotope shifted matrices enable the use of low mass ion precursor scanning for targeted metabolite identification

**DOI:** 10.1186/1477-5956-9-2

**Published:** 2011-01-17

**Authors:** Charles B Reilly, Sri H Ramarathinam, Nicholas A Williamson, Anthony W Purcell

**Affiliations:** 1 Department of Biochemistry and Molecular Biology, The Bio21 Molecular Science and Biotechnology Institute, The University of Melbourne, Parkville, Victoria, 3010 Australia

## Abstract

We describe a method to identify metabolites of proteins that eliminates endogenous background by using stable isotope labeled matrices. This technique allows selective screening of the intact therapeutic molecule and all metabolites using a modified precursor ion scan that monitors low molecular weight fragment ions produced during MS/MS. This distinct set of low mass ions differs between isotopically labeled and natural isotope containing species allowing excellent discrimination between endogenous compounds and target analytes. All compounds containing amino acids that consist of naturally abundant isotopes can be selected using this scanning technique for further analysis, including metabolites of the parent molecule. The sensitivity and selectivity of this technique is discussed with specific examples of insulin metabolites identified within a complex matrix using a range of different validated low mass target ions.

## Background

Mass spectrometry (MS) can identify molecules based on their distinctive mass and/or on the mass of product ions generated by fragmentation of the parent ion. It is this sensitivity and selectivity that is often exploited to distinguish a target peptide from a mixture of other closely related species [1]. The common approach for peptide identification is to use characteristic fragment ions produced during relatively mild collision activated dissociation of parent ions. The series of "sequencing" ions produced differ in mass by the residue mass of specific amino acids in addition to various neutral losses and intramolecular reactions. These ions then enable the *de novo *reconstruction of the primary structure of the parent peptide ion from the spectrum of fragment ions. This systematic approach relies on the prediction the fragmentation pattern of the peptide, however many variables such as collision energy and sequence composition influence fragmentation, often making it difficult to predict ion intensity relationships. This can then lead to challenges in the interpretation and prediction of MS/MS spectrum [2-5]. The uncertainty in how a particular peptide may fragment and the reliance of a classical "sequencing ions" approach to peptide identification may in some cases lead to the incomplete assignment of the spectra, where sequencing ions provide an internal sequence "tag" but fail to provide enough information for a complete peptide sequence. This is especially the case when the data from which peptide identities are made is of poor quality due to very low abundance of the parent species, or if there is only a small number of MS/MS ions present that can be used for sequencing. These sequencing difficulties are further compounded when attempting to identify peptides in very complex samples.

We propose that stable isotope labelling can improve the certainty of peptide identity. Stable isotope labelling amino acids in cell culture (SILAC), where the "heavy" isotope (^13^C, ^15^N etc.) is used for quantification of proteins by MS, is now a well-established, safe and reproducible technique [6]. The advantage of SILAC is that the only chemical difference is a single neutron that alters the atom and subsequently the amino acid mass, while not altering its biological relevance. There have also been significant advances in the complete incorporation of stable isotopes into multicellular organisms such as mice, with heavy isotope mouse feed now commercially available [6,7]. Thus the scope of experiments that incorporate stable isotope labelling is broad and can range from simple experiments in prokaryotes through to complex experiments in genetically modified mice. In this study we demonstrate how stable isotope labelling can be used for the discovery and identification of peptide metabolites. In contrast to similar studies where the protein or biotherapeutic is modified by incorporation of a stable isotope [8,9], we propose the inverse, where the entire matrix is modified instead of the target protein. Complete incorporation of, in our case, ^15^N into the matrix of interest allows discrimination between matrix-derived metabolites and peptides derived from the target protein.

In addition to validating peptide identity, differential labeling can be combined with triple quadrupole technology to enable targeted data acquisition, whether in the form of multiple reaction monitoring (MRM) or during precursor ion scanning. The former technique is extremely sensitive and is currently being exploited for quantitation of known compounds; however, MRM requires knowledge of the precursor mass of the analyte which in the case of metabolites is often unknown. In contrast, precursor ion scanning can be performed without prior knowledge of the precursor mass. Combining this with the knowledge of a specific and distinguishing fragment ion can enable the use of "information dependant acquisition" (IDA) to reveal the full MS/MS of target compounds. We describe how, by using a stable isotope labeled matrix, we are able to employ the precursor ion scan to detect molecules liberated from insulin. Here the shifted matrix results in the otherwise "normal" fragment ions of the insulin becoming the unique and distinguishing features that can be harnessed to trigger targeted MS/MS analysis.

The target ions used in this case are low mass ions that are below 200 *m/z*; these ions include, but are not limited to, the immonium and related ions and can be indicators for the presence of corresponding unlabeled amino acid residues (Table 1). Currently low mass ions are under-exploited in peptide identification; however, recent studies such as Hohmann *et al *[10] suggest that the immonium ion can be used as a quantitative indicator. The presence and relative intensity of the immonium ion reflect not only the presence of a particular amino acid residue but can also describe where within the peptide the residue is located. In our study we are particularly interested in the immonium ion due to its relationship to peptide amino acid composition. The immonium ion can form through various context-dependant mechanisms but consists of the general structure RCH = NH_2_^+ ^with R being the side chain of the amino acid residue. This results in a defined set of immonium ions (and related ions that have undergone an additional fragmentation in the form of a neutral loss) that represent all of the 20 common amino acids. The particular feature of these ions that has been exploited in this study is that the immonium ions always contain at least one nitrogen as a remnant from the amide bond found in all peptides. This has meant that with the complete incorporation of ^15^N in a metabolic matrix, the immonium ions derived from endogenous peptides found within the matrix will not be observable at the normal *m/z*.

**Table 1:** List of low mass target ions in native and stable isotope labelled peptides

Residue	Native Immonium ion	Related native ions	^15^N Immonium ion
Alanine, A	44		45
Arginine, R	129	59,70,73,87,100, 112	133
Asparagine, N	87	70	89
Apartic acid, D	88	70	89
Cysteine, C	76		77
Glutamic acid, E	102		103
Glutamine, Q	101	56,84,129	103
Glycine, G	30		31
Histidine, H	110	82,121,123, 138,166	113
Isoleucine, I	86	44,72	87
Phenylalanine, F	120	91	121
Proline, P	70		71
Leucine, L	86	44,72	87
Lysine, K	101	70,84,112,129	103
Methionine, M	104	61,70	105
Serine, S	60		61
Threonine, T	74		75
Tryptophan, W	159	77,117,130,132, 170,171	161
Tyrosine, Y	136	91, 107	137
Valine, V	72	41,55,69	73

**Insulin tryptic peptides**		**Target ions used for precursor scanning**	

FVNQHLCGSHLVEALYLVCGER		120 (F), 70 (P/R/N), 86 (L/I), 72 (V)	
GFFYTPKT		120 (F), 70 (P/R/N)	
GIVDQCCTSICSLYQLENYCN		72 (V), 86 (L/I), 70 (P/R/N)	

(Top) List of immonium and related ions for the 20 naturally abundant amino acids and the expected isotopic shifts of immonium ions when 15N labeled. (Bottom) Tryptic Insulin digest peptides and the potential target ions to be used for information dependant MS/MS acquisition (IDA) targets during a precursor ion scan.

**Figure 1:**
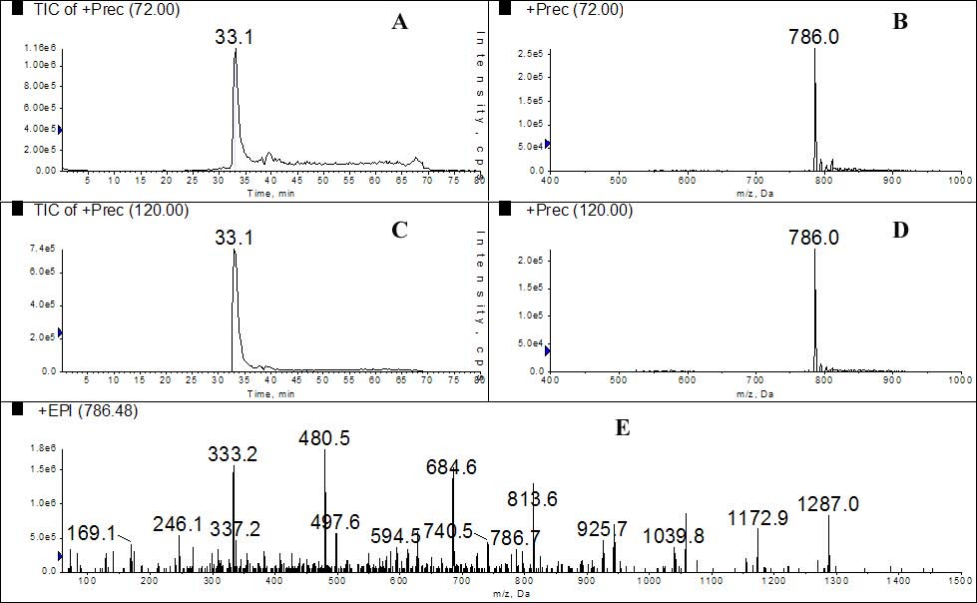
**Multiple precursor ion scanning for GluFib from within a complex mixture**. 1 pmol of GluFib was added to 40 μg ^15^N matrix consisting of the acid soluble fraction of *E. coli*. A double precursor ion scan was performed using the immonium ion targets of (A) 72 (valine) and (C) 120 (phenylalanine). Figure 1, shows the precursor ion scans both detect the 786 *m/z *ion (B and D respectively) and subsequently trigger IDA of a product ion spectra (E) that produces sequencing ions for the GluFib peptide EGVNDNEEGFFSAR.

During this study we tested the ability to selectively screen out specific peptides from a ^15^N complex mixture using immonium ions corresponding to amino acid residues found within a target peptide. Figure 1 shows the well characterized Glu Fibrinopeptide EGVNDNEEGFFSAR (GluFib) being selectively screened from a complex mixture. Here 1 pmol of GluFib is added to a complex matrix consisting of 40 μg ^15^N compounds taken from the acid soluble fraction of *E. coli *which had been cultured exlusively in ^15^N media. A multiple precursor ion scan that simultaneously monitors for more than one target ion (in this case two) is employed to target the MS analysis towards GluFib. The target ions used are 72 and 120 and represent the amino acid residues of valine and phenylalanine respectively. During this analysis the entire matrix was ignored and signal detection from the precursor scan was only obtained from unlabeled GluFib, thus triggering a targeted MS/MS While at 1 pmol concentration it is possible to identify GluFib within this matrix using a conventional LC-MS/MS approach, the differential isotope labeling enables high confidence during characterization with conventional sequencing. In addition, only MS/MS spectra belonging to GluFib is provided, reducing the data load for search algorithms.

**Figure 2:**
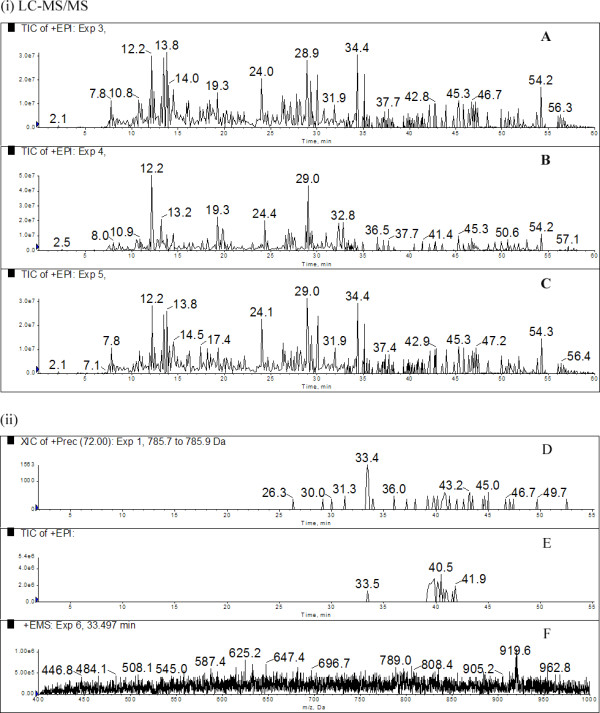
**Precursor ion scanning for GluFib from a ^15^N complex mixture below the detection level of a traditional LC-MS/MS approach**. 100 fmol of GluFib was spiked into 80 μg (total protein) of a ^15^N matrix consisting of the acid soluble fraction of *E. coli *and split into two. One half was analysed as a regular LC-MS/MS (i) while the other as a Precursor scan for *m/z *72 valine (ii). At this concentration of GluFib the regular LC-MS/MS did not select the target peptide for MS/MS due to the complexity of the sample. In contrast the precursor scan identified GluFib and automatically selected it for MS/MS data acquisition. The LC-MS/MS experiment consisted of an IDA that selects the three most intense ions from each mass spectrum for product ion acquisition. (i) Shows the TIC of all three product ion spectra acquired during the single LC-MS/MS experiment, none of which selected the precursor mass of 786 *m/z *(GluFib) for product ion acquisition due to the complexity of the sample. In contrast the precursor scan (ii) shows the product ion spectra TIC (E) with a peak at 33.5 mins, the precursor ion triggering IDA at this time point had an *m/z *786, corresponding to GluFib and appropriately product ion spectra was acquired for confirmation. (F) The total mass spectrum corresponding to the time point when GluFib was detected and triggered product ion data acquisition, highlighting the low abundance of the GluFib *m/z *786 ion relative to the complex background. (D) Extracted ion chromatogram (XIC) for the GluFib precursor mass from the precursor scan for *m/z *72 showing the most intense signal during the elution of GluFib. (The precursor ion scan chromatograms are cropped to highlight the time when GluFib is eluted.)

The real advantage of this strategy is in situations where target molecules coelute with many compounds of higher intensity. We therefore directly compared the targeted precursor scan data acquisition to untargeted LC-MS/MS where GluFib was titrated into a ^15^N complex mixture (40 μg ^15^N as above). In Figure 2 (i, A-C)) we show the total ion chromatogram (TIC) of all three product ion spectra acquired as a result of IDA during a single LC-MS/MS when 50 fmol of GluFib is added to the complex mixture. At this concentration GluFib is completely ignored and not selected for product ion acquisition during untargeted LC-MS/MS. In contrast, when the same sample was run with identical LC and ion source conditions, but with a precursor ion scan monitoring for an immonium ion for valine (72 *m/z*), GluFib was efficiently detected. The TIC for the precursor scan in Figure 2 (ii, D-F) shows a strong and distinct signal when GluFib is present despite the enhanced mass spectrum (EMS) that was obtained during IDA showing low abundance of the target ion (785.7 amu, Figure 2F), relative to all other ions present. During this analysis no signal was obtained and thus no product ion spectra acquired until the time point 33.4 min, which is consistent with the highest concentration of GluFib during the chromatographic elution when an extracted ion chromatogram is performed on the precursor ion scan (Figure 2D).

Furthermore, the selection of the internal residues valine and phenylalanine illustrates the strength of the approach through its ability to employ a variety of low mass ions that can come from residues found throughout the target peptide.

Lastly this targeted approach to metabolite identification is especially relevant to the monitoring and discovery of biotherapeutic metabolites in complex matrices where targets are of unknown precursor mass. Here we took peptides derived from a tryptic digest of injectable insulin and added them to the same complex matrix mentioned above (40 μg ^15^N as above). We observed specificity and selectivity for peptides liberated from insulin by virtue of proteolysis by bacterial peptidases contained within the *E. *Coli lysate. The same sample was also run as a standard LC-MS/MS experiment and both results submitted to a ProteinPilot version 4 search algorithm. While the LC-MS/MS came back with no positive identifications for insulin, the precursor scan identified the predicted tryptic peptides as well as additional unexpected insulin peptides. These included peptides with modified cysteine residues and truncated peptides that were the result of residual protease activity within the ^15^N *E*. *coli *matrix.

**Figure 3:**
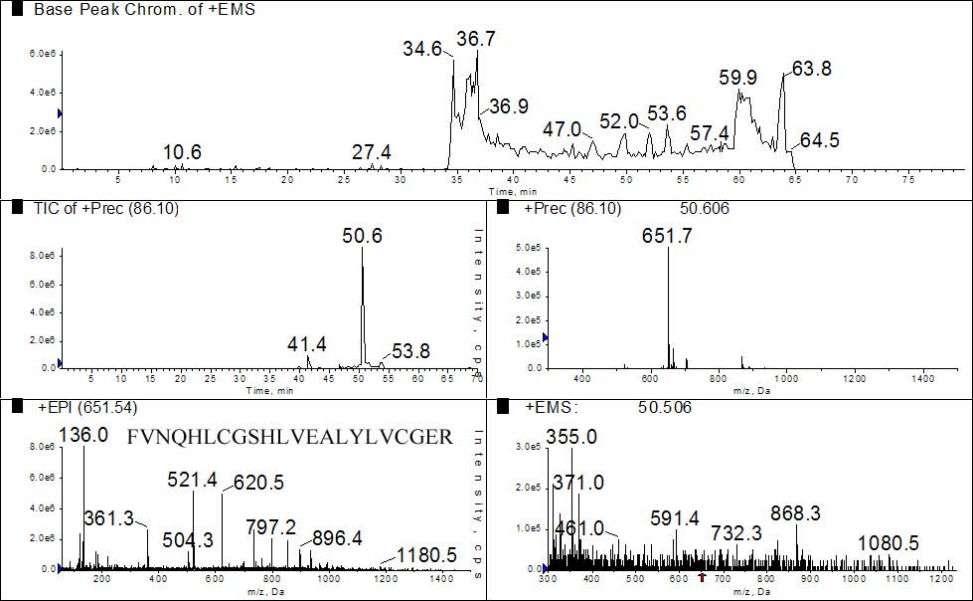
**Insulin peptides were combined with a ^15^N complex matrix**. 2 pmol of an insulin tryptic digest was added to 40 μg (total protein) of a ^15^N matrix, consisting of the acid soluble fraction of *E. coli*. The same sample was run as a regular LC-MSMS experiment and as precursor ion scanning experiments. Target ions during the precursor scans consisted of immonium and related ions corresponding to residues L, I, Y, V, N, D and F. (A) The base peak chromatogram during the LC-MS/MS procedure. (B) The total ion chromatogram (TIC) for the precursor ion scan with target ion 86 *m/z *which corresponds to Ile and Leu. (C) The precursor scan at time point 50.6 min (which corresponds to the major peak in the TIC) showing the presence of an intense peak for 651.5 *m/z *which corresponds to the insulin peptide FVNQHLCGSHLVEALYLVCGER. (D) The mass spectrum at the 50.6 min time point showing that the 651.5 *m/z *ion is of low abundance relative to the complex spectral background. (E) The Product ion spectrum for the ion at *m/z *651.5 which was acquired due to detection of a target ion during the precursor scan at time point 50.6 min. The sequencing ions lead to a positive identification of the peptide FVNQHLCGSHLVEALYLVCGER. Both the LC-MS/MS and precursor ion scan data sets were submitted to the same ProteinPilot version 4 search engine. Only the Precursor ion scan identified peptides liberated from insulin.

The experimental cycle loop of the 4000 QTrap instrument used consisted of precursor ion scans that targeted up to four specific immonium ions, with the detection of any one of these ions prompting IDA. This in turn resulted in acquisition of an enhanced product ion spectrum and an EMS. Figure 3 shows a total ion chromatogram (TIC) for the precursor scan for *m/z *86 (immonium ion corresponding to leucine/isoleucine) and a major peak at time point 50.6 min. At this time point the major precursor mass that was giving a signal for the presence of an 86 *m/z *was 651.7 and accordingly an IDA-triggered EPI was generated. The MS/MS data in this spectrum corresponds to that of the insulin-derived tryptic-like peptide FVNQHLCGSHLVEALYLVCGER (Table 2). In addition to the EPI we included an EMS to be triggered during the precursor scan in order to provide context. Figure 3 (bottom right panel) shows the mass spectrum at the same time point for when the 651 *m/z *peptide was identified (50.6 min). Here we see that the peptide would not have been observed during a conventional MS/MS acquisition due to the large number of ions with much greater ion intensity than the insulin peptide signal. These data shows that despite the target peptide being in low abundance relative to the complex spectral background it still gives the highest intensity signal during the precursor scan, resulting in "targeted data acquisition" for a peptide that was derived from insulin regardless of matrix complexity. When the data acquired was subjected to analysis by ProteinPilot version 4 the only successful protein ID was insulin.

**Table 2:** Sequencing ions for the insulin peptide FVNQHLCGSHLVEALYLVCGER.

b ions	y ions
148.1	2601.3
247.1	2454.2
361.2	2355.1
489.2	2241.1
626.3	2113.1
739.4	1976.0
899.4	1862.8
956.4	1702.9
1043.5	1645.8
1180.5	1558.8
1293.6	1421.7
1392.7	1308.7
1521.7	1209.6
1592.8	1080.6
1705.8	1009.5
1868.9	896.4
1981.1	733.4
2081.1	620.3
2241.1	521.2
2298.1	361.2
2427.2	304.2
2583.3	175.1

## Summary

We have shown that by using stable isotope labeled matrices combined with precursor ion scanning and monitoring low mass ions, we are able to selectively screen for peptides liberated from proteins of interest. The precursor scanning technique assumes no prior knowledge or prediction of how the products within that matrix will form and is able to specifically screen peptides that may even contain unanticipated post translational modifications. The selectivity employed in triple quadrupole technology allows for targeted fragmentation data acquisition for the peptides/metabolites of interest at low abundance relative to a complex endogenous background. Furthermore, the data acquired is readily searchable using current informatics algorithms without a requirement to analyze MS/MS data containing ^15^N or alternative stable isotopes. This technique will find many applications in monitoring changes to biomolecules - especially in relation to biotherapeutics - within complex sample mixtures. Moreover, with the advent of isotopically labeled animal feed one can envision the use of this technique for absorption, distribution, metabolism, and excretion (ADME) type studies of peptide and protein drugs in experimental animals.

## Competing interests

The authors declare that they have no competing interests.

## Authors' contributions

CBR performed the majority of the experiments, conceived the study, contributed to experimental design and drafted the manuscript. SHR provided technical assistance and mass spectrometry support. NAW provided expert mass spectrometry guidance. AWP conceived the study, participated in it design and coordination and co-wrote the manuscript. All authors read the final manuscript.
